# Techno-economic potential of bioethanol from bamboo in China

**DOI:** 10.1186/1754-6834-6-173

**Published:** 2013-11-29

**Authors:** Jade Littlewood, Lei Wang, Colin Turnbull, Richard J Murphy

**Affiliations:** 1 Department of Life Sciences, Imperial College London, London SW7 2AZ, UK; 2 Centre for Environmental Policy, Imperial College London, London SW7 1NA, UK; 3 Centre for Environmental Strategy, University of Surrey, Guildford, Surrey GU2 7XH, UK

**Keywords:** Bamboo, Bioethanol, Advanced biofuel, Lignocellulose, Pretreatment, Saccharification, Techno-economic, Minimum ethanol selling price, China

## Abstract

**Background:**

Bamboo is potentially an interesting feedstock for advanced bioethanol production in China due to its natural abundance, rapid growth, perennial nature and low management requirements. Liquid hot water (LHW) pretreatment was selected as a promising technology to enhance sugar release from bamboo lignocellulose whilst keeping economic and environmental costs to a minimum. The present research was conducted to assess: 1) by how much LHW pretreatment can enhance sugar yields in bamboo, and 2) whether this process has the potential to be economically feasible for biofuel use at the commercial scale. Pretreatments were performed at temperatures of 170-190°C for 10–30 minutes, followed by enzymatic saccharification with a commercial enzyme cocktail at various loadings. These data were then used as inputs to a techno-economic model using AspenPlus™ to determine the production cost of bioethanol from bamboo in China.

**Results:**

At the selected LHW pretreatment of 190°C for 10 minutes, 69% of the initial sugars were released under a standardised enzyme loading; this varied between 59-76% when 10–140 FPU/g glucan of commercial enzyme Cellic CTec2 was applied. Although the lowest enzyme loading yielded the least amount of bioethanol, the techno-economic evaluation revealed it to be the most economically viable scenario with a production cost of $0.484 per litre (with tax exemption and a $0.16/litre subsidy). The supply-chain analysis demonstrated that bioethanol could be economically competitive with petrol at the pump at enzyme loadings up to 60 FPU/g glucan. However, in a prospective scenario with reduced government support, this enzyme loading threshold would be reduced to 30 FPU/g glucan.

**Conclusions:**

Bioethanol from bamboo is shown to be both technically and economically feasible, as well as competitive with petrol in China. Alternative approaches to reduce bioethanol production costs are still needed however, to ensure its competitiveness in a possible future scenario where neither tax exemptions nor subsidies are granted to producers. These measures may include improving sugar release with more effective pretreatments and reduced enzyme usage, accessing low cost bamboo feedstock or selecting feedstocks with higher/more accessible cellulose.

## Background

The urgency for development of sustainable liquid biofuels in the transport sector is recognised globally due to concerns regarding energy security, oil price volatility and environmental pollution [1]. In 2011, China contributed to 29% of world carbon dioxide emissions, and therefore it has significant potential to influence the present and future global energy situation [2]. Currently, almost half of China’s oil consumption is imported, and with the projection that demand for fossil fuel oil will reach 250 million tons by 2030, it is crucial for the China to consider biomass alternatives as part of their renewable energy plan [3,4]. In 2009, the number of private cars owned in China exceeded the United States, resulting in it being the world’s largest auto market. Establishment of a biofuel industry in China is therefore an attractive solution to manage the problems of environmental pollution, energy independence and rural development within the transport sector [3,5,6].

In its development of biofuel policy, China’s 10th five-year plan (2001–2005) proposed a biofuel industry to utilise surplus grain stocks. Through the government’s support for biofuel production, China has become the third largest bioethanol producer in the world after the US and Brazil, with an overall fuel ethanol production capacity of 1.9 million tons in 2008 [7]. Now, approximately 10% of the total liquid fuel supply is accounted for by biofuels, and there has been an increase in pilot plant projects cropping up in Henan, Anhui, Jiangsu and other provinces. However, concerns regarding food security resulted in the government’s order to halt construction of corn-based plants and promote non-food feedstocks which can be grown on marginal and abandoned lands instead [3]. The Ministry of Agriculture has estimated that marginal and abandoned land area for energy crops in China ranges from 35–75 million hectares, of which 24 million hectares are cultivable, thereby suggesting a significant land area for growing biofuel crops [8]. However the lack of a key non-food feedstock that can be grown on such lands is the major constraint on the expansion of fuel ethanol production in China [9].

While bamboos are used by 2.5 billion people worldwide for applications ranging from food to construction to paper, a novel purpose for it in the field of bioenergy has been proposed in more recent years [10]. These fast growing, resilient, perennial grasses have been shown to thrive in diverse climatic and soil conditions and to possess numerous desirable traits for biofuel production [10-12]. Bamboo resources in China are amongst the richest in the world. More than 500 different bamboo species occur (36% of the world total) and China is regarded as the epicentre of bamboo origin and distribution worldwide [13,14]. China’s bamboo forests cover 7.6 million hectares of land across 18 provinces and are located mainly in the Southern region of the Yangtze River drainage basin [13-15]. The largest commercial applications include shoot production for food, culms for material uses and as a raw material for pulping [16]. Since 1970, China’s bamboo sector has increased by 54%, and the total forest area has grown at annual rate of 3% since 1980 [17].

As a member of the Graminae family, the composition of bamboo is highly similar to other grasses utilised for biofuel purposes (e.g. switchgrass, Miscanthus). Its cell wall is comprised of the polymeric constituents cellulose, hemicellulose and lignin. The complex physical and chemical interactions between these components prevent enzymes from readily accessing the microfibrillar cellulose during the saccharification stage of its conversion into biofuel [18,19]. As a result of this recalcitrance, a pretreatment stage is needed to maximise hydrolysis of cell wall sugars into their monomeric form [18,20,21]. Numerous pretreatments, grouped into chemical, physical, physico-chemical and biological types have been shown to successfully improve sugar release from different feedstocks. While the technologies are varied, most aim to achieve solubilisation of lignin and/or hemicellulose, reduce cellulose crystallinity, increase biomass surface area and disrupt cell wall component interactions [22-24]. One effective pretreatment uses hot water at high temperature and pressure to solubilise hemicellulose as a route to enhance enzyme accessibility to cellulose [22]. Due to the lack of chemical requirement, Liquid hot water (LHW) pretreatment has been shown to be attractive from both economic and environmental standpoints. Furthermore, by keeping the reaction pH between 4 and 7, there is minimal formation of sugar degradation products, which are known to be toxic to downstream fermentative microorganisms [25,26].

The aim of this work was to explore the techno-economic potential for establishing a bamboo-to-bioethanol industry in China. Various pretreatment and saccharification conditions were investigated to identify the optimal conditions for maximising sugar release from the bamboo feedstock. These conditions were used as inputs for the techno-economic modelling to yield a production cost of bioethanol under different scenarios. A supply chain analysis was then used to assess whether the price of bioethanol sold at the pump under the defined conditions could be competitive with petrol in China.

## Results and discussion

### Bamboo material

The chemical compositions of raw (non-pretreated) *Phyllostachys dulcis* and *Phyllostachys viridiglaucescens* bamboo species were not significantly different and were averaged to use as a baseline value (referred to as “raw material” in this study) for comparison with pretreated material. The composition of raw bamboo had a moisture content of approx. 10% and a total sugar content of 64.2% of dry matter (DM). Of this, the predominant sugar was glucan (38.4%) followed by xylan (20.5%), galactan (3.6%) and arabinan (1.8%). Lignin, extractives and ash comprised 20.8%, 13.5% and 0.9% of DM, respectively. An acetyl group of approximately 3.0% of DM is reported to be common for most bamboo species [27]. After enzymatic saccharification the total sugar release from the non-pretreated material was 7.2% of DM, equivalent to 11.3% of the theoretical maximum sugar release.

### Screening of liquid hot water pretreatment conditions

**Figure 1:**
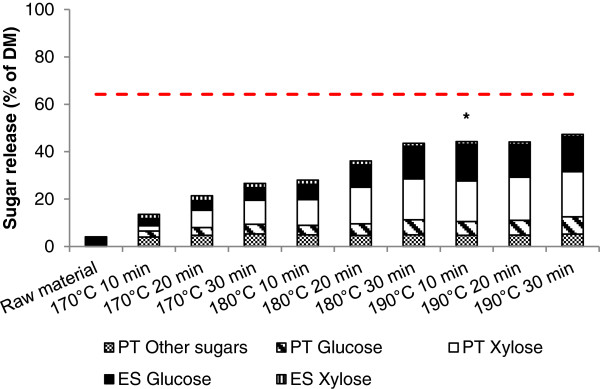
**Sugar release from pretreatment and enzymatic saccharification as a percentage of DM (PT – Pretreatment, ES – Enzymatic saccharification, other sugars refer to sum of galactose and arabinose).** The red dashed line indicates the theoretical maximum in raw material (64.2% of DM). * Selected LHW pretreatment condition.

The total sugar release from both pretreatment and enzymatic saccharification are summated to assess the efficacy of pretreatment on releasing cell wall sugars. The pretreatment sugar yields include glucan and xylan as well as galactan and arabinan solubilisation (referred to as “other sugars” in Figure 1) into the liquid hydrolysate during pretreatment, and these are assumed to be in monomeric form. The enzymatic saccharification sugar yields comprise glucose and xylose release from the residual glucan and xylan in the pretreated biomass. The total sugar yield is expressed as a percentage of the original feedstock DM (64.2% is the theoretical maximum sugar yield from the raw bamboo).

After LHW pretreatment, total sugar release from the different conditions ranged from 13.6% to 47.3% of DM (21.2% to 73.7% of the theoretical maximum). There was no significant difference between sugar release from LHW pretreatment at 190°C for 10, 20 or 30 minutes (ANOVA, p > 0.05). Therefore a total yield of 44.3% of DM (69.0% of the theoretical maximum, equivalent to over a 6-fold increase from raw material) by LHW pretreatment at 190°C for 10 minutes was selected for further experiments. Under these conditions, 84% of the initial xylan was released during pretreatment, and 47% of the glucan from the pretreated material was released during enzymatic saccharification. Interestingly, while the maximum pretreatment xylose release was achieved at the 190°C for 30 minutes pretreatment (93% of initial xylan), this did not correspond to the highest glucose release during saccharification. Instead, glucose release was maximised during pretreatment at 190°C for 10 minutes. This indicates that the additional xylan removal achieved during the more severe pretreatment did not effectively enhance glucan accessibility during saccharification after a certain level, and furthermore suggests that factors other than xylan content may be significant in hindering enzymatic conversion of glucan at this stage.

It is evident that in general the more severe pretreatment conditions (up to 190°C) resulted in greater xylan (and hemicellulose) solubilisation and also increased glucose release during enzymatic saccharification (Figure 1). These results therefore support the theory that solubilisation of xylan during pretreatment has a substantial effect on improving glucan accessibility in enzymatic saccharification, and is one indicator of a successful LHW pretreatment [18]. Our findings are similar to the results of García-Aparicio et al. [28] who found a 55.8% improvement in glucan conversion of bamboo after a steam pretreatment and suggest that a hydrothermal pretreatment such as LHW can substantially improve sugar release in bamboo. Nevertheless, it should also be borne in mind that while many studies show this linear relationship between xylan removal and glucan digestion, it is unlikely that xylan can be selectively removed without disrupting other biomass components. Therefore it cannot be concluded whether increased glucan accessibility can be exclusively attributed to selective xylan removal or is a result of a combination with other factors. Finally, although for the modelling we assumed that solubilised xylan was present as monomeric xylose and available for fermentation, several studies have demonstrated that LHW-solubilised xylan is mainly oligomeric rather than monomeric [29,30]. While the genetic modification of one *Geobacillus* strain has been demonstrated and patented [31] to ferment oligomers directly into bioethanol, most other fermentative microorganisms require an additional hydrolysis step to convert sugars into monomers or small oligomers for fermentation.

### Enzymatic saccharification of LHW pretreated bamboo

**Figure 2:**
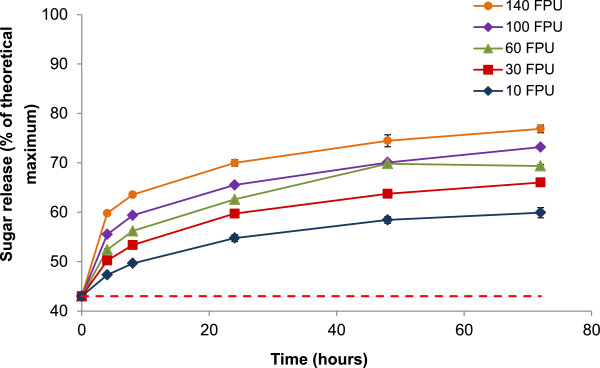
**Total sugar release from pretreatment and enzymatic saccharification as a percentage of the theoretical maximum after 72 hours from LHW pretreated bamboo (190°C for 10 minutes) treated with five enzyme loadings.** Red dashed line represents sugar release under pretreatment at 190°C for 10 minutes (43.0%). Error bars represent standard error (n = 3).

With the selected LHW pretreatment condition (190°C for 10 minutes), Cellic Ctec2 (a commercial enzyme cocktail from Novozymes A/S Denmark) was applied at five loadings ranging from 10-140 FPU/g glucan to generate scenarios for the techno-economic analysis. Due to the importance of enzyme cost to the overall process economics for lignocellulosic bioethanol production, it has been suggested that decreasing enzyme loadings in the conversion process is a key target for process optimisation [32]. Therefore the lower enzyme loadings were applied to assess whether this could be reduced whilst maintaining a sufficiently high level of sugar release. Higher enzyme loadings were also applied to investigate whether sugar yields could be maximised by saturating the pretreated biomass with enzyme. Sugar release (from pretreatment and enzymatic saccharification combined) is expressed as a proportion of the theoretical maximum to demonstrate potential improvements in sugar yield under enzyme loadings of 10, 30, 60, 100 and 140 FPU/g glucan (Figure 2). The total solubilisation of glucan, xylan, galactan and arabinan into monomeric sugars during pretreatment was equivalent to 43.0% of the theoretical maximum (shown by the red dashed line in Figure 2). This was measured by compositional analysis before and after pretreatment to determine the content of polymeric cell wall sugars; the difference between these values represents the proportion of sugars that were hydrolysed into monomers during pretreatment. The first time point taken at 4 hours is therefore equal to 43.0% plus the additional glucose and xylose release during enzymatic saccharification.

After 72 hours, although sugar release ranged from 59% to 76% of the theoretical maximum, there was no significant improvement with incremental increases in applied enzyme loadings. These improvements were even less at higher loadings, suggesting that despite being subjected to an effective pretreatment, a portion of the cell wall remained resistant to enzymatic hydrolysis. These findings are consistent with those reported by Cara et al. [33] for olive tree biomass for example, which showed that after pretreatment a significant portion of cellulose remained recalcitrant to enzymes even at high enzyme dosages. This also reinforces the idea that hemicellulose removal in bamboo is effective but only up to a certain point, after which alternative routes may be required to fully maximise release of the remaining cell wall sugars.

### Techno-economic analysis – effect of enzyme loading on bioethanol production from bamboo using LHW pretreatment

**Figure 3:**
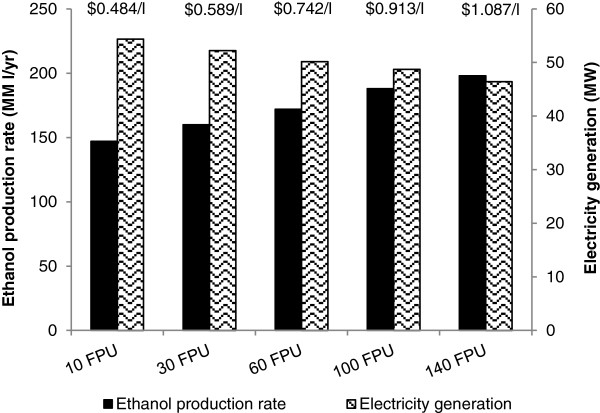
**Ethanol production, electricity generation and minimum ethanol selling prices (MESPs) for LHW-pretreated bamboo treated with five enzyme loading scenarios.** MESP values listed above bars.

The techno-economic analysis showed that bioethanol production ranged from 147 to 198 million litres per year, and electricity generation ranged from 46 to 54 megawatts (MW), depending on the enzyme loading applied (Figure 3). Greater enzyme use resulted in higher sugar release, and therefore increased ethanol production with a concomitant decrease in electricity generation due to a reduced flow of residual biomass to the combustion area. Interestingly, even though bioethanol was the main product of this process, a greater level of production did not lead to lower bioethanol cost due to the high cost of enzyme required to achieve these yields. As a result, bamboo pretreated with LHW for 10 minutes at 190°C and saccharified with 10 FPU/g glucan of Cellic CTec2 led to the lowest minimum ethanol selling price (MESP) of $0.484 per litre. The increasing MESPs with enzyme loading demonstrated that the cost of purchasing additional enzyme to release cell wall sugars outweighed the benefit of producing more bioethanol. This finding differs from a study by Macrelli et al. [34] on sugarcane bagasse and leaves, who showed that doubling the enzyme dosage resulted in a MESP reduction of 12% due to a corresponding 33% increase in bioethanol production. The discrepancies can be attributed to specific sugar yield results, which demonstrated that doubling the enzyme loading only improved total sugar release by approximately 7% in our results with bamboo.

Our results showed that with LHW-pretreated bamboo, the additional benefit of adding more enzyme to improve yields was smaller than the cost of purchasing this enzyme. As a result, simply producing less bioethanol was a more economically advantageous scenario. This conclusion however is highly dependent on the enzyme cost. In this study, a price from Kazi et al. [35] was adopted, which was also estimated to be at the high end compared with other economic analyses. However, most of the prices of enzyme cocktails for large-scale cellulosic bioethanol production are unknown and based on hypothetical price projections. Alternatively, authors sometimes use a “top-down” measure by reporting in dollars per litre, which is an aggregate assumption that fails to take into account variation in enzyme (e.g. loading and actual cost) [35,36]. Nonetheless, this issue is highly debated and is recognised to be an inconsistent parameter which seriously hinders the robustness of techno-economic models [36]. Assuming that enzyme loading is a key barrier to reduce the MESP, one way to minimise costs would be to simply apply less enzyme thereby compromising bioethanol production. Other approaches which research is focusing on include: 1) identifying and optimising pretreatments to improve biomass accessibility during saccharification, 2) advanced development (breeding etc.) to have a higher cellulose content or reduced lignin content/composition, or 3) selection and breeding of naturally-occurring genotypes which are shown to be more amenable to enzymatic hydrolysis [37-39].

### MESP cost breakdown analysis

**Figure 4:**
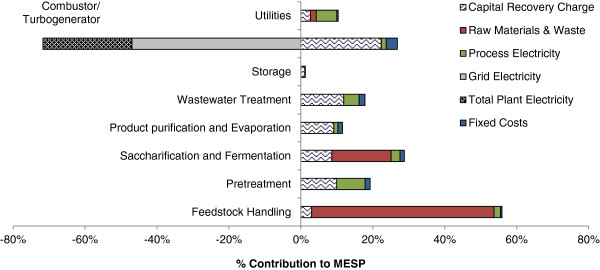
MESP cost breakdown analysis for bioethanol from bamboo using LHW pretreatment with a 10 FPU/g glucan enzyme loading.

A cost breakdown analysis of the 10 FPU/g glucan enzyme scenario revealed the leading cost contributors to the MESP in the bamboo to bioethanol process (Figure 4). The three highest positive cost contributors were bamboo raw materials and waste (51%, shown in the feedstock handling area), enzyme in the saccharification & fermentation area and capital expenditure in the combustion/turbogeneration area. The contribution of enzyme varied from 17% to 68% of the MESP depending on the loading scenario. While the minimum loading of 10 FPU/g glucan resulted in the lowest MESP, it still comprised almost one-fifth of the production cost, demonstrating the significant contribution that this parameter has within the bioethanol conversion process. The combustor/turbogeneration area had the highest capital cost amongst the different areas and accounted for 22% of the MESP. Despite these hefty capital costs, the combustor area had a negative cost of −45% due to significant credits gained from the export of surplus electricity from the combustion of bamboo residues that were not converted into bioethanol.

Despite the relatively low bamboo prices of approximately $45/tonne used in this analysis, the feedstock handling area still had the largest contribution to the MESP (56% of the total), which seems to be a common trend in techno-economic evaluations of biomass-to-bioethanol pathways [40-42]. While the number of natural bamboo forests in China may be able to provide sufficient amounts of biomass to support a bioethanol industry of this scale, in reality, many of these resources would be diverted towards production of higher value products. Historically, Chinese factories used to purchase whole bamboo culms and were forced to deal with large amounts of wasted residues. A solution for this problem resulted in the “pre-processing bamboo revolution” which involves separating culms into different sections for various supply chains as an approach for potential utilisation of 100% of the material with zero waste [43]. The three largest bamboo sectors currently include handicrafts, bamboo shoots and industrial processing [43]. The industrial processing sector is further divided into sub-sectors ranging from low-value products such as paper and pulp, to high-value products such as flooring and laminated furniture [43]. Bamboo prices are based on its size and part of the culm, and reflect its potential end product. This study has taken into account these price differences and has adopted a bamboo cost of waste material, which is one of the lowest amongst different sectors and would benefit the MESP. Even so, feedstock cost still represents the single largest cost contributor to the MESP, demonstrating the significance of selecting low-cost feedstocks for bioethanol production economics.

**Figure 5:**
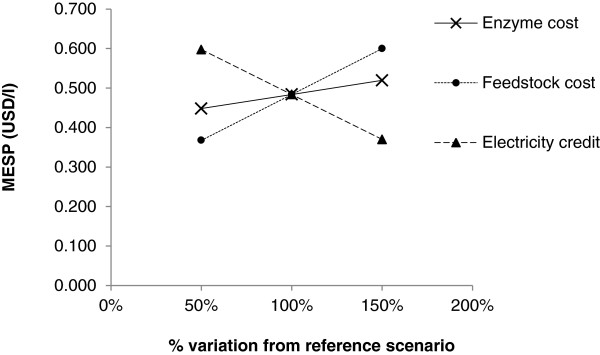
Sensitivity analysis of MESP with a +/−50% variation in the price of feedstock, enzyme and electricity credit from the reference scenario.

It is evident that at respective contributions of 51%, 45% and 17% of the MESP, feedstock cost, enzyme cost and price of renewable electricity are major economic determinants influencing the price of bioethanol from bamboo. Therefore, a sensitivity analysis for the 10 FPU/g glucan enzyme scenario was performed to analyse the impact of these parameters on the MESP. Each parameter was varied by a range of 50% from the baseline cost used in the reference scenario, based on sensitivity reports from the literature, typically ranging from 20–50% of the original cost value [35,44-46]. Feedstock cost was varied between $22.3-$66.9/dry tonne; enzyme cost ranged between $253.5-$760.5/tonne; and electricity credit was manipulated between $0.056-$0.167/kWh (Figure 5). The gradient of the slope indicates the influence of these parameters, such that a steeper slope has a greater effect on the results and vice versa with a smaller slope. Lines increasing from left to right show a positive correlation between the parameter and the MESP, and the reverse for lines decreasing left to right.

Both feedstock and enzyme cost are positively correlated with the MESP, whereas electricity credit is negatively correlated. Therefore, higher enzyme and feedstock prices result in greater MESPs and conversely, lower electricity prices result in an increase in the MESP. The slope of feedstock cost is the highest at 0.232, and at a +/−50% variation in price, the MESP ranges from $0.368-$0.600/litre (Figure 5). Electricity credit generates the second highest slope of (−)0.227 and MESP values vary between $0.370-$0.597/litre. The MESP is least sensitive to enzyme cost with a slope of 0.071, and ranges from $0.448-$0.519/litre. These figures support the cost breakdown results stating that the MESP is most sensitive to feedstock cost followed by electricity credit and then to enzyme cost. It is inevitable that these cost assumptions are dependent on the local situation and can vary at any time, whether this is due to market price fluctuations or to changes in government regulations. Therefore understanding the extent to which this can affect the price of bioethanol production is valuable information for all relevant stakeholders.

### Competitiveness of bioethanol with petrol at the pump in China

**Figure 6:**
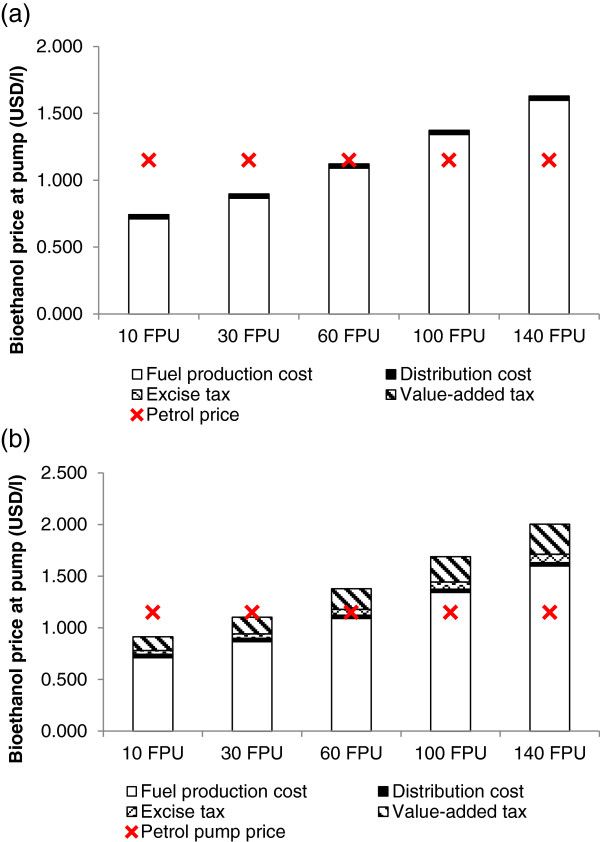
China bioethanol pump price for five enzyme loading scenarios in (a) 2011 with a 16 cent per litre subsidy and fuel excise and value-added tax exemptions, and (b) a prospective future scenario with no form of government support measures.

A theoretical bioethanol pump price was generated based on the reference year 2011 to examine whether the bamboo-to-bioethanol process could be competitive with petrol in China. The pump price includes the fuel production cost, a distribution cost ($0.032/litre), value-added tax (17%) and a fuel excise tax (5%) [42,47,48]. The energy content of bioethanol is less than petrol such that 0.68 litres of petrol is equivalent to 1 litre of bioethanol. The bioethanol prices have been adjusted to their petrol equivalent for comparison in Figure 6(a) and (b).

In 2011 and up till now, bioethanol production in China receives significant levels of government support in order to make it commercially feasible. Since 2001, after establishment of the fuel ethanol industry, various measures of support have been implemented to incentivise fuel ethanol production in China. Fuel ethanol producers and blenders as well as gasohol (fuel blend of ethanol and gasoline) retailers are exempted from the national consumption tax and value-added tax, and designated producers can also receive a subsidy of $0.16/litre bioethanol [47]. Under these conditions, bamboo bioethanol pump prices at enzyme loadings of 10 to 60 FPU/g glucan scenarios would be competitive with petrol in 2011 (Figure 6(a)). Therefore, amongst these conditions the 60 FPU/g glucan scenario is considered to be the maximum or “threshold” enzyme level before bioethanol becomes uncompetitive with petrol.

Government support in China for bioethanol is currently high and includes both exemption from VAT and fuel excise tax, and subsidy. However, this subsidy which was originally $0.20 per litre in 2008, has been progressively scaled back each year [47]. It is expected that future levels of support will diminish, so the cost of bioethanol production will need to be reduced in order to remain competitive with petrol. A prospective scenario was therefore developed to assess a possible future where neither tax exemptions nor subsidies are granted to producers to determine the conditions under which bamboo bioethanol could still be competitive with petrol (Figure 6(b)). In this projection, the “threshold” enzyme loading was reduced from 60 FPU/g glucan to 30 FPU/g glucan; whereby enzyme dosages greater than 30 FPU/g glucan were no longer able to compete with petrol based on 2011 prices.

## Conclusion

A techno-economic assessment was used to evaluate the potential for producing bioethanol from bamboo using liquid hot water pretreatment under various pretreatment and saccharification conditions. A LHW pretreatment at 190°C for 10 minutes was selected as the optimal condition for maximising sugar release which reached 69% of the theoretical maximum after 72 hours of saccharification. Under this condition a greater proportion of sugar was released during pretreatment compared with saccharification, whereby the predominant sugars were xylose and glucose in pretreatment and saccharification, respectively. Enzymatic saccharification with five loadings (10–140 FPU/g glucan) of Cellic CTec2 led to a total sugar release ranging from 59–76% of the theoretical maximum. Little improvement was found in total sugar release despite significantly increasing enzyme loading, and even at the highest dosage a portion of cellulose (about 20%) remained resistant to enzymatic hydrolysis.

The economic analysis revealed that the lowest enzyme loading had the most commercially viable scenario (MESP of $0.484/litre) even though it produced the least amount of bioethanol and generated the greatest level of co-product electricity. This was primarily due to the significant enzyme contribution to cost, which at higher loadings was not defrayed adequately by an increase in the amount of sugar released. A cost breakdown and sensitivity analysis of the 10 FPU/g glucan scenario demonstrated that the cost of raw materials was the greatest contributor, with bamboo and enzyme purchase accounting for 51% and 17% of the MESP, respectively. The combustion area was also a significant contributor due to the reduced level of bioethanol production in this scenario, and had an overall contribution of −45% of the MESP. The supply-chain model showed that bamboo would be competitive with petrol at the pump in scenarios with enzyme loadings of 60 FPU/g glucan and lower. However the prospective scenario, which made the assumption of no tax breaks or subsidy, demonstrated that lower enzyme loadings would still permit bioethanol from bamboo to maintain its economic competitiveness with petrol under the technical conversion efficiencies modelled.

## Methods

### Plant material and preparation of biomass

*Phyllostachys dulcis* and *Phyllostachys viridi*-*glaucescens* bamboo culms (estimated to be about 5 years of age) were harvested from Kew Gardens in London. Branches and leaves were removed and each culm was left to air-dry for 2 weeks. Full culms were ground using a Retsch AS2000 cutting mill with a 1 mm screen then sieved to collect material between the size 850 and 180 μm. By oven-drying biomass samples at 105°C, dry matter (DM) and therefore moisture contents could be calculated.

### Compositional analysis

For raw (non-pretreated) bamboo material, a two-step extraction step using water followed by 95% ethanol was performed according to the NREL LAP protocol “Determination of extractives in biomass [49] using a Dionex® Accelerated Solvent Extractor (ASE) 200. Samples were air-dried, re-weighed and moisture contents calculated to determine the percentage extractives.

Compositional analysis for raw bamboo material as well as pretreated material was based on the NREL LAP protocol “Determination of structural carbohydrates and lignin in biomass” [50]. Polymeric carbohydrates are hydrolysed into monomeric forms and measured by HPLC using a Bio-Rad Aminex HPX-87P column at 80°C with a flow rate of 0.6 mL/min water mobile phase on an Agilent 1200 series HPLC. The lignin fractionates into acid-soluble and acid–insoluble material which is assayed by UV–vis spectroscopy and gravimetric analysis, respectively (along with ash content).

### Enzymatic saccharification

Prior to enzymatic saccharification, enzyme activity was measured according to the NREL protocol “Measurement of cellulase activities” [51]. This determined the cellulase activity in terms of “filter paper units” (FPU) per millilitre of original enzyme solution. Cellic CTec2 protein weight was calculated to be approximately 183 mg/mL (1.10 mg/FPU of enzyme) [52]. Two rounds of enzymatic saccharifications were performed. The first was a standardised saccharification on raw (unpretreated) and pretreated bamboo material, which was used to assess the effect of pretreatment on glucose and xylose release. This followed the protocol of Selig et al. [53], and was carried out for 72 hours using an enzyme loading of 60 FPU/g glucan of a cellulase enzyme mixture containing a 1:1 ratio of Celluclast 1.5 L and Novozyme 188. The selected conditions for LHW pretreatment were based on maximising sugar yields, and were subjected to a second round of enzymatic saccharification using the commercial Cellic® CTec2 enzyme from Novozymes A/S, Denmark. CTec2 contains a blend of cellulase, β-glucosidase and hemicellulose enzymes, and is an enzyme mixture designed for commercial use and is therefore considered to be a realistic enzyme option for the techno-economic model [54]. A time course assay with CTec2 was performed with loadings of 10, 30, 60, 100 and 140 FPU/g glucan and samples were harvested at 4, 8, 24, 48 and 72 hours. Glucose and xylose concentrations were assessed by HPLC as described above.

### Liquid hot water pretreatment

LHW pretreatment was carried out using the Dionex ASE 200 machine. The ASE is typically used for performing biomass extraction, but has been used for pretreatments and was adapted here [55,56]. The machine operates by pumping the solvent through a cell containing the biomass sample. The cell is heated for the desired amount of time by an oven until the pretreatment has been completed. The solvent is then moved from the cell to a vial collecting the liquid fraction, and the remaining biomass solid fraction is left inside the cell [57]. Biomass (2.0 g DM) was pretreated in triplicate under the conditions shown in Table 1. Following pretreatments, biomass was air-dried overnight and mass loss and moisture content measurements were made the next day. Only the water-insoluble solids from pretreatment were carried through for subsequent enzymatic saccharification.

**Table 1:** Liquid hot water pretreatment conditions

**Parameter**	**Value**
Temperature (°C)	170, 180, 190
Time (min)	10, 20, 30
Pressure (psi)	500
Heat-up time (min)	7, 8, 9
Flush volume	100%
Purge time (sec)	120

### AspenPlus™ process design and simulation

**Figure 7:**
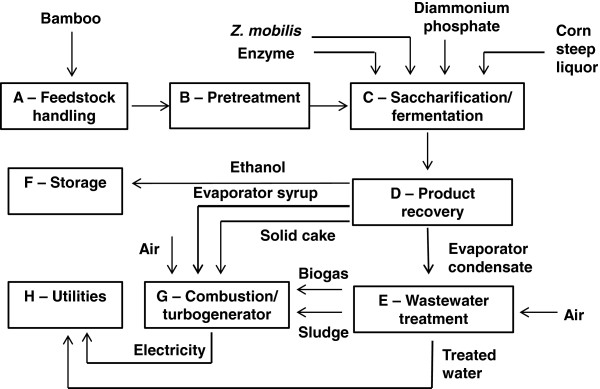
Schematic diagram of bamboo-to-bioethanol process in AspenPlus™.

The techno-economic process design was adapted from the NREL model [41], and is designed to process 2,000 dry metric tonnes of bamboo biomass per day, operating at 8,410 hours per year. An overview of the main process areas is shown in the schematic diagram in Figure 7.

Bamboo is unloaded at the feedstock handling (Area A in Figure 7) where it is washed, then milled to a suitable particle size. It is then conveyed to pretreatment (Area B) where it undergoes LHW pretreatment at a total solids loading of 30% (w/w) [41]. Pretreated bamboo is sent to separate saccharification and fermentation (Area C) where material is first enzymatically hydrolysed into monomeric sugars and then fermented into ethanol using the bacterium, *Zymomonas mobilis*. This microorganism was selected based on the study by NREL, who have research experience using this recombinant *Z*. *mobilis* strain with the ability to simultaneously co-ferment glucose and xylose into ethanol [41]. Other studies have also demonstrated that *Z*. *mobilis* is acid tolerant and can grow over a wide pH range from 3.5 to 7.5, and recent research has isolated a strain more tolerant to commonly encountered inhibitors during biomass fermentation [58-60]. Saccharification is carried out at 50°C for 72 hours. The hydrolysate is cooled to 32°C and sent to two *Z*. *mobilis* seed inoculation trains with a residence time of 24 hours each, as well as fermentation tanks operating for 36 hours. The strain of *Z*. *mobilis* used is a recombinant microorganism fermenting both hexose and pentose sugars. Nutrient loadings of corn steep liquor (CSL) and diammonium phosphate (DAP), and the fermentation sugar conversion efficiencies (95% of glucose, 85% of xylose and arabinose) are adopted from the NREL process [41]. Of the monomeric sugars, it is assumed that 3% are converted into glycerol, succinic acid and xylitol as a result of contaminations [41]. The fermentation beer is sent to product recovery (Area D) where ethanol is concentrated through distillation and molecular sieve adsorption to 99.6%. Distillation bottoms from the distillation column (containing unfermented monomeric sugars, organic acids and solid residues such as lignin, extractives and ash) are sent to a series of evaporators to produce a condensed syrup and a lignin-rich solid cake. These are then sent to the combustor/turbogenerator (Area G) for steam and electricity generation.

Wastewater treatment includes anaerobic and aerobic digestion which treats and recycles used water to reduce the total amount discharged to the environment and the purchased fresh water requirement. In anaerobic digestion, 91% of organic matter is converted into microorganism cell mass and biogas. The biogas with a composition of 51% CH_4_/49% CO_2_ (w/w) is assumed to be produced at a yield of 228g biogas per kg COD (chemical oxygen demand) removed [41]. Treated water is next cleaned in aerobic digestion, where 96% of the remaining soluble organic matter is removed.

The concentrated syrup and solid cake from the distillation is combined with the biogas and cell mass (sludge) from wastewater treatment to be fed to the combustor (Area G) for Combined Heat and Power (CHP) generation. High-pressure steam is extracted from the turbine to meet process heat requirements. Generated electricity supplies the process energy demand, and any surplus electricity is sold to the National Grid as a co-product credit.

The utilities area (Area H) includes the cooling tower, plant air and clean-in-place systems. The storage area (Area F) is used to store bamboo material, chemicals, and products.

### Cost assumptions

Mass and energy balances were generated in AspenPlus™ software. The Total Capital Investment (TCI) was determined from purchased and installed equipment costs. Equipment costs were derived from NREL’s vendor quotations, which were scaled up or down according to the exponential scaling expression [41]:

(1) $$\text{New}\;\text{cost}=\left(\text{Base}\;\text{cost}\right)\;{\left(\frac{\text{New}\;\text{size}}{\text{Base}\;\text{size}}\right)}^{f\;\text{scale}}$$

All costs in this study were indexed to the reference year of 2011. Direct and indirect costs were summated to yield the TCI. Direct costs included warehouse, site development and additional piping, comprising 4%, 9% and 4.5% of the Inside-battery-limits (ISBL) equipment costs (Areas B-D involved in production of bioethanol), respectively. Indirect costs included prorateable costs (10% of total direct cost), field expenses (10%), home office and construction (20%), project contingency (10%) and other costs (10%) [41].

The raw material costs (Table 2) contributed to the variable operating costs and were only incurred while the process was in operation. Fixed operating costs included labour and various overhead items and were incurred whether or not the plant was producing at full capacity. Annual maintenance materials were estimated as 3% of the ISBL capital cost. Local property tax and property insurance were assumed to be 0.7% of the fixed capital investment [41].

**Table 2:** Summary of raw material costs

**Materials/chemicals/energy**	**Price ($/tonne)**	**Reference**
Bamboo	44.6	[61]
Corn steep liquor (CSL)	57.9	[41]
Diammonium phosphate (DAP)	502.5	[62]
Enzyme	507.0	[35]
Sorbitol	1148	[41]
Fresh water	0.26	[41]
Boiler feed water chemicals	4268.6	[41]
Cooling tower chemicals	3636.9	[41]

Other China-specific cost parameters (Table 3) involved in the analysis were included feedstock cost, waste disposal charges, electricity credit and income tax. The number of employees was been adopted from Humbird et al. [41], baseline salaries were derived from a personal communication with a chemical processing plant in China, and labour ratios for each country were calculated according to the average salary of each country [63].

**Table 3:** Summary of cost and fuel price parameters (2011) in China

	**China**	**Ref**
Fuel production price parameters		
Delivered feedstock price	44.6	[61]
Transportation cost ($/tkm)	0.05	[64]
Landfill tax ($/t)	4.5	[65]
Electricity credit^a^ ($/kWh)	0.11	[66]
Income tax	25%	[67]
Fuel pump price parameters		
Pump price in 2011	1.240	[68]
Distribution cost ($/litre)	0.032	[69]
Fuel excise tax	5%	[47]
Value-added tax (VAT)	17%	[47]
Subsidy	16 cents/litre	[47]

^a^Credit refers to the amount that renewable electricity generators can receive from selling their excess electricity to the grid or other suppliers/distributors.

### Discounted cash flow analysis

Once the TCI and operating costs were determined, the minimum ethanol selling price (MESP) was determined using a discounted cash flow analysis. This is the bioethanol price generated using a discount rate of 10%, at which the net present value of the project is zero. This model is based on an ‘n^th^-plant’ assumption. This eliminates additional costs associated with pioneer plants by assuming other plants using the same technology are currently in operation [41]. The discounted cash flow analysis parameters are listed in Table 4.

**Table 4:** Discounted cash flow analysis parameters

**Parameters**	**Value**
Plant life	30 years
Discount rate	10%
Financing	40% equity
Loan terms	10-year loan at 8% APR
General plant depreciation	200% declining balance^a^
General plant recovery period	7 years
Steam plant depreciation	150% declining balance
Steam plant recovery period	20 years
Construction period	3 years
0–12 months	8% of project cost
12–24 months	60% of project cost
24–36 months	32% of project cost
Working capital	5% of fixed capital investment
Start-up time	3 months
Revenues during start-up	50%
Variable costs incurred during start-up	75%
Fixed costs incurred during start-up	100%

^a^Depreciation method is the IRS Modified Accelerated Cost Recovery System (MACRS).

### Supply chain model

A supply-chain model was established to determine the bioethanol price at pump for comparison with petrol in 2011. This price includes the bioethanol production cost, fuel excise tax, value-added tax (VAT), a feedstock transportation cost and a fuel distribution cost. The energy content of bioethanol (21.2 MJ/l) is less than petrol (31.2 MJ/l); 1 litre of bioethanol is therefore equivalent to 0.68 litres of petrol. It was assumed that bamboo is transported by lorry from a distance within 50 km of the bioethanol plant. An average distribution cost of $0.032/litre of bamboo was adopted from Slade et al. [69,70].

## Abbreviations

DM: Dry matter; LHW: Liquid hot water; FPU: Filter paper unit; MESP: Minimum ethanol selling price; VAT: Value-added tax; DAP: Diammonium phosphate; CSL: Corn steep liquor; COD: Chemical oxygen demand; CHP: Combined heat and power; NREL: National renewable energy laboratory; ISBL: Inside-battery-limits; TCI: Total capital investment.

## Competing interests

The authors declare that they have no competing interests.

## Authors’ contributions

JL and RJM designed the study. JL carried out the experimental analysis and drafted the manuscript. JL and LW performed the process design and economic evaluation. CT and RJM supervised the work and edited the manuscript. All authors read and approved of the final manuscript.
